# The Role of WHO Surgical Checklists in Reducing Postoperative Adverse Outcomes: A Systematic Review

**DOI:** 10.7759/cureus.70923

**Published:** 2024-10-06

**Authors:** Shehrbano Qaiser, Maham Noman, Muhammad Sheharyar Khan, Umer Waseem Ahmed, Aamna Arif

**Affiliations:** 1 Surgical Emergency, Oxford University Hospitals NHS Foundation Trust, Oxford, GBR; 2 Surgery, University Hospitals Birmingham NHS Foundation Trust, Birmingham, GBR; 3 Renal and Transplant, Oxford University Hospitals NHS Foundation Trust, Oxford, GBR; 4 Obstetrics and Gynaecology, West Suffolk Hospital NHS Trust, Bury St Edmunds, GBR; 5 Rehabilitation Medicine, North Bristol NHS Trust, Bristol, GBR

**Keywords:** mortality, post operative complication, surgical safety, surgical safety checklist, who surgical safety checklist

## Abstract

Surgical safety remains a critical aspect of modern healthcare, particularly as the number of surgical procedures continues to rise, placing greater demands on resources and increasing the potential for errors. In response to this challenge, various mitigation strategies have been implemented to improve operative outcomes. One such strategy, introduced by the WHO in 2008, is the Surgical Safety Checklist. Despite its widespread adoption globally, its acceptance remains limited in developing countries. This systematic review aimed to evaluate the impact of the WHO Surgical Safety Checklist, specifically the sign-in, time-out, and sign-out components, on reducing post-operative adverse effects in surgical patients. A single-step search strategy was employed across multiple databases, including Medline, CINAHL, Embase, Cochrane Database, ProQuest, Index Copernicus, Google Scholar, and Scopus. Additionally, reference lists of identified reports and articles were manually searched to identify further relevant studies. Only studies published in English before September 2022 that focused exclusively on the WHO Surgical Safety Checklist were included. Studies on other checklists or those with confounding factors, such as international surgical outcomes studies, were excluded from this analysis. After screening 17,821 publications based on their titles and abstracts, 93 studies met the initial inclusion criteria and underwent full retrieval and assessment for methodological quality. Ultimately, 13 studies were deemed of sufficient quality to be included in the review. Among these, 10 studies reported outcomes related to complication rates, with nine of them demonstrating a decrease in complication rates following checklist implementation. Similarly, 13 studies reported outcomes related to mortality rates, with 12 reporting a decrease in mortality rates associated with checklist use. In conclusion, the application of the WHO Surgical Safety Checklist has been shown to improve surgical outcomes by reducing post-operative adverse effects, including mortality and complication rates. However, further research is warranted to assess the checklist's impact on the quality of life of surgical patients, which would contribute to enhancing its overall acceptability. Continued investigation into these areas will help further strengthen the evidence supporting the widespread adoption and effective implementation of the WHO Surgical Safety Checklist across diverse healthcare settings globally.

## Introduction and background

Surgical complications are a significant concern for the surgical team, as any post-operative sequelae can render their efforts futile [1]. Common surgical complications include post-operative sepsis, wound infection, anastomotic leak, drain bypassing, bleeding from the surgical site, operating on the wrong patient or the wrong surgical site, and even death. The efficacy of the surgical team is therefore largely dependent on patient outcomes [2].

Many mitigation strategies have been implemented to reduce surgical complications. These include the introduction of aseptic measures, the use of prophylactic antibiotics, improving teamwork and communication, ensuring the reliability of machinery and disposal equipment, improving glycemic control, maintaining post-operative normothermia, identifying comorbid conditions, improved scrutiny in selecting surgical patients, improving the efficiency of the scrub team, and the appropriate use of pneumatic devices [3]. Additionally, the selection process for patients undergoing surgery has improved, allowing for a better assessment of surgical risks and overall patient suitability. Furthermore, enhancements in scrub team efficiency, such as meticulous swab counts, have contributed to minimizing the risk of retained surgical items and ensuring a safer surgical environment.

Among the global health challenges faced by healthcare facilities, preventing potential harm from surgery is one that is less appreciated. Surgeons have worked immensely hard over the years to improve surgical techniques and procedures. However, relatively less attention has been paid to implementing checklists to avert post-operative complications [4]. The global volume of deaths caused by post-operative complications is estimated to be 4.2 million per year [5]. Deaths are preventable with sufficient measures in addition to those previously mentioned. Adverse events in the postoperative period can often be traced back to the level of adherence to established guidelines and practices, particularly the lack of proper communication between patients and caregivers [6].

In addition, there has been a significant change in the population trends of patients receiving surgical treatment over the past decade. Increased survival rates and improved healthcare have substantially increased the number of aging patients with multiple comorbidities opting for and being eligible for surgery [7]. Consequently, there is an inevitable overall increase in complication rates among these patients. Considering all these factors, it is sufficient to say that surgical complications are increasing over time, and previous mitigation strategies are somewhat insufficient to deal with the current scenario [8].

The introduction and implementation of surgical safety checklists have been relatively underappreciated in reducing the adverse effects of surgery. In 2009, the WHO introduced a 19-item checklist aimed at bringing structure to surgical procedures by improving communication among team members and promoting mutual understanding [9]. The primary purpose of the checklist is to ensure a good practical approach by the surgical team at three different intervals: sign-in (before induction of anesthesia), time-out (before skin incision), and sign-out (before the patient leaves the operating room).

Despite the potential benefits of the checklist, multiple barriers exist in its implementation. One such barrier is the acceptability and trust of hospitals in this checklist [10]. While it is widely accepted in developed countries, developing countries still struggle with understanding its implementation. This is partly due to the heavy patient load and limited resources in these regions [11]. The existing burnout among nursing staff leads to resistance against adding such a checklist. Even if checklists are implemented, poor applicability is often expected due to dynamic educational and social factors, such as ambiguity and confusion about the checklist's purpose and negative attitudes toward its adoption [12]. In some settings, the checklist may be applied, but resource limitations render some items, such as the use of a pulse oximeter, pointless to the team [13].

Other checklists have also been recommended for widespread use globally. The SURgical PAtient Safety System (SURPASS) checklist is one of the methods used for improving patient outcomes in surgical patients. However, this checklist is relatively extensive, covering preoperative, peri-operative, and postoperative items. Consequently, outcomes are influenced by multiple stages of intervention, making it difficult to analyze peri-operative factors in isolation [14]. Similarly, the Universal Checklist has been subject to regional modifications, preventing uniformity in its application [15].

Due to variable international results on the usage of peri-operative checklists and the lack of published reviews in the past six years, it is imperative to revisit their utility. For this review, the checklist proposed by WHO in 2009 was considered, as it focuses primarily on peri-operative factors [9]. The purpose of this investigation is to provide a contemporary and globally representative review of the usage of peri-operative checklists and systematically appraise the evidence addressing the question: "Does the WHO Surgical Safety Checklist prevent postoperative adverse effects in patients undergoing surgical procedures?".

## Review

Materials and methods

This systematic review was conducted following the guidelines of the Joanna Briggs Institute systematic review process. A search strategy was formulated based on defined inclusion and exclusion criteria, with modifications made during the review process to refine the primary selection criteria. Additionally, search outcomes and methodological criteria were included. The central question for the review was the following: “Are WHO surgical checklists (sign-in, time-out, and sign-out) effective in reducing post-operative adverse outcomes?”.

A systematic literature search was conducted for studies published from 2009 to 2022. Relevant studies meeting the selection criteria were included in the review. The databases searched were Medline, CINAHL, Embase, Cochrane Database, Proquest, Index Copernicus, Google Scholar, and Scopus.

The keywords used in the search strategy included the following: "checklists," "WHO Surgical Safety Checklist," "postoperative complications," "postoperative mortality," "mortality," "time in," "time out," and "sign in." The keyword "mortality" yielded the highest number of searches, with 786,906 hits, while "WHO Surgical Safety Checklist" produced 17,821 hits. Since the focus of this review was to include articles reporting outcomes in terms of postoperative complications and mortality, studies with other objectives were excluded. The search terms used in this review are detailed in Table 1.

**Table 1:** Search terms used and the corresponding number of hits.

Search line number	Search term used	Number of results
1	Checklist	39263
2	WHO Surgical Safety Checklist	17821
3	1 OR 2	40116
4	Postoperative complications	61555
5	postoperative complications	54930
6	4 OR 5	68462
7	"postoperative mortality"	8348
8	"mortality"	786906
9	7 OR 8	786914
10	Time in	57481
11	Time out	44972
12	Sign in	18551
13	10 OR 11 OR 12	62891

Inclusion/Exclusion Criteria

This study included studies published in English or translated to English before September 2022. Eligible study designs were randomized controlled trials, quasi-experimental studies, cohort studies, and case-control studies focusing on operative outcomes where the WHO surgical safety checklist was implemented. Only the WHO surgical safety checklist was considered for comparability and homogeneity. Primary outcomes were defined as mortality, while secondary outcomes included the incidence of complications.

Complications considered included surgical site infection (SSI), non-SSI wound infections, sepsis, septic shock, myocardial infarction, pulmonary embolism, respiratory failure, internal bleeding, and deep vein thrombosis if directly caused by the surgical procedure.

Search Outcomes

Initially, the search strategy yielded 786,914 hits. After screening according to the PICO (population, intervention, control, and outcomes) question, 17,821 relevant studies were identified. Automation and duplicate removal across databases excluded 16,340 studies. An additional 1,246 studies were excluded based on keywords, leaving 235 studies. After the title review, 142 studies were excluded, resulting in 93 studies for abstract and full-text analysis. Ultimately, 93 studies were included in this study.

Methodological Quality

The 93 studies meeting the inclusion criteria underwent critical analysis. Studies were assessed for statistical analysis methods, study design appropriateness, relevance of reported outcomes, and journal reliability. Descriptive studies were evaluated using the Joanna Briggs Institute Checklist, before/after and non-randomized control trials with the ROBINS-1 checklist, and case-control and cohort studies with the SIGN and CASP study checklists, respectively.

**Figure 1:**
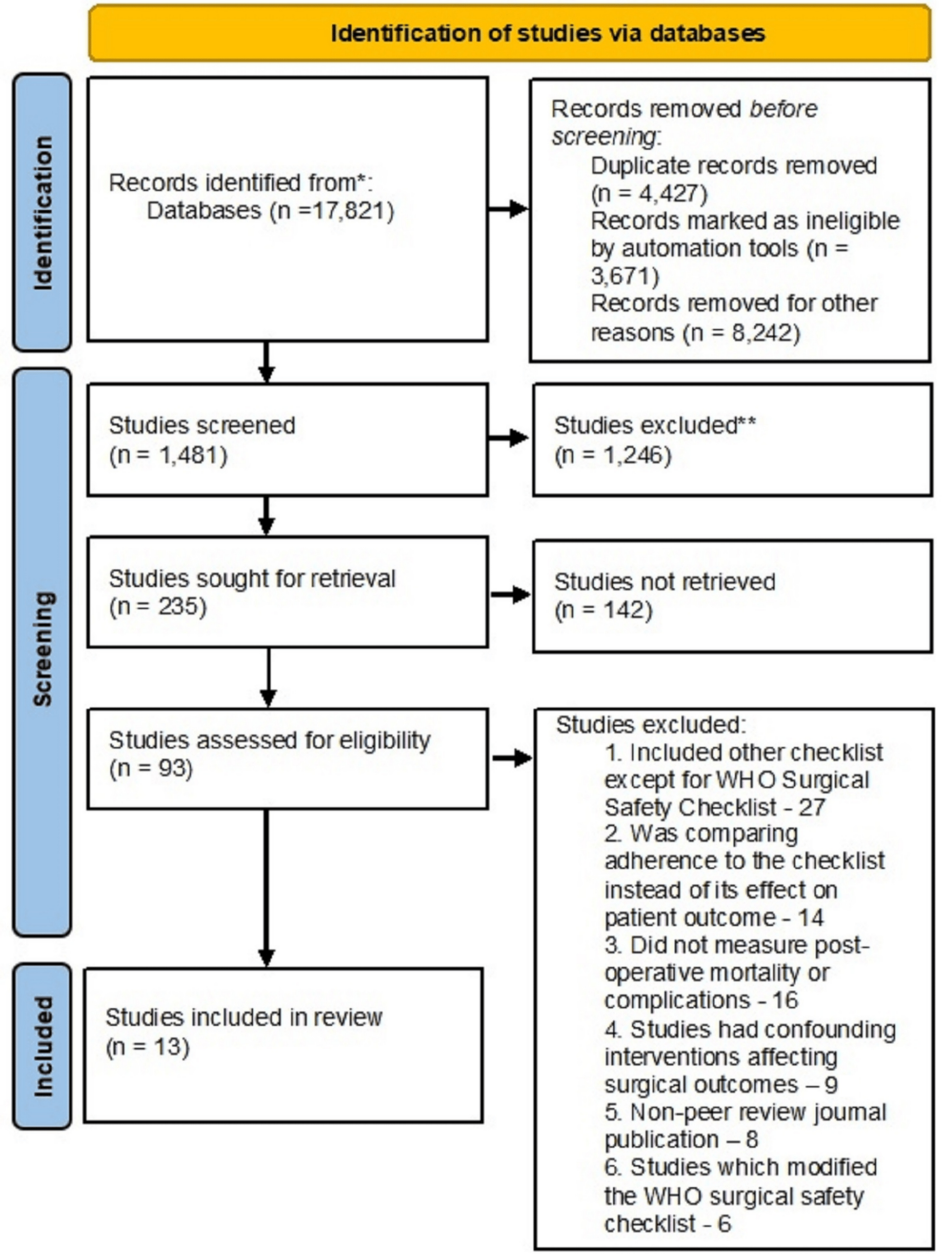
Search strategy and outcomes. Preferred Reporting Items for Systematic Reviews and Meta-Analyses (PRISMA) Flowchart

After scrutiny, 13 studies were included in the final analysis. Exclusions were primarily due to inappropriate methodologies, including 27 studies that used checklists other than WHO's, and six studies that modified the WHO checklist to suit their settings (see Figure 1).

Results

This systematic review included a total of 13 studies that evaluated patient outcomes following the implementation of the WHO surgical safety checklist. Details of the appraisal can be found in Table 2.

**Table 2:** Systematic appraisal.

Experimental (Intervention) Study								
Authors, date & country	Study design	Participants, recruitment & sampling methodology	Intervention		Outcome measures	Results		Comments
Askarian et al., 2011 Iran [16]	Interventional Study Before/After	Non-randomized, Non probability consecutive sampling technique. The study site was a 374-bed referral educational hospital in Shiraz, Iran, with 6 operating rooms. The study lasted 6 months. Total of 294 patients were included, out of which 150 were pre-implementation and 144 were post implementation.	The checklist covered 3 surgical stages. Persons included were operating room team members. Checklist was presented to the surgeons, nursing staff, OT assistants		Rates of postsurgical complication before and after application of the surgical safety checklist underwent comparison. The complications were measured objectively using a structured questionnaire.	Incidence of any complication before and after intervention was 22.9% and 10% (P = .03). Five checklist items were in total compliance. The most common complication was surgical site infection. Implementation of the checklist, responsibility in 2 stages, such as time out and sign out, were significant (P < 0.05).		The study only included patients who underwent gastrointestinal surgery and had relatively small sample size of only 300 patients.
Haynes et al., 2009 USA [17]	Interventional Study Before/After	Prospectively collected data on clinical processes and outcomes from 3733 consecutively enrolled patients 16 years of age or older who were undergoing noncardiac surgery. We subsequently collected data on 3955 consecutively enrolled patients after the introduction of the Surgical Safety Checklist.	World Health Organization's Safe Surgery Saves Lives program		The primary end point was the rate of complications, including death, during hospitalization within the first 30 days after the operation.	The rate of death was 1.5% before the checklist was introduced and declined to 0.8% afterward (P=0.003). Inpatient complications occurred in 11.0% of patients at baseline and in 7.0% after introduction of the checklist (P<0.001)		The study included all non-cardiac surgery patients. The study had a very adequate sample size and more than 10 pan-continental settings were involved.
Lubbeke et al., 2013 Switzerland [18]	Interventional Study Before/After (Quasi-experimental)	Prospectively data was collected on the outcomes of the patients which included a comparison between two groups. One group (609 patients) was prior to the intervention and the second group (1818 patients) was after the intervention. Consecutive sampling	Checklist was applied in 2009 in 27 operating room and was supervised by resident working officers		The outcomes included in this study were unplanned return to the operating room for SSI treatment, unplanned return to the operating room for any reason, unplanned admission into the ICU and in hospital mortality.	Unplanned return to the operating room was 7.4% vs 6.0% post intervention, reoperation for SSI was 3.0% vs 1.7% post intervention, admission into the ICU was 2.8% vs 2.6% and in hospital mortality rate was 4.3% vs 5.8% post intervention.		The study had a relatively small sample size which made the results relatively unreliable compared to other studies that have been conducted.
Urbach et al., 2014 Canada [19]	Interventional Study Before/After	Two groups were compared in a total of 133 hospitals. Before the checklist 109.341 patients were included and after implementation of checklist a total of 106,370 patients were included. Consecutive sampling	Adoption of WHO surgical checklist in all acute care hospitals in Ontario Canada		Compared operative mortality, rate of surgical complications, length of hospital stay, and rates of hospital readmission and emergency department visits within 30 days after discharge among patients undergoing a variety of surgical procedures	The adjusted risk of death during a hospital stay or within 30 days after surgery was 0.71% before implementation of a surgical checklist and 0.65% afterward (P=0.13). The adjusted risk of surgical complications was 3.86% before implementation and 3.82% afterward.		Implementation of surgical safety checklists in Ontario, Canada, was not associated with significant reductions in operative mortality or complications.
Moore et al., 2021 Australia [20]	Interventional Study Before/After	Analysed 9475 patients in the 18-month period before the checklist and 10,589 afterwards. Consecutive sampling	Implemented the World Health Organization surgical safety checklist at Auckland City Hospital from November 2007		Compared postoperative mortality and increase days alive and out of hospital, both measured to 90 postoperative days.	Mean number of days alive and out of hospital in the cohort after checklist implementation was 1.0 days longer than in the cohort preceding implementation, p < 0.001. Ninety-day mortality was 4% and 3% in the cohorts before and after checklist implementation, p = 0.40.		Patients experienced improving postoperative outcomes from 2004 to 2013, including the periods before and after implementation of the surgical checklist.
Observational Study								
Authors, date & country and focus of study	Study design	Participants, recruitment & sampling methodology		Exposure – disease or risk factors	Outcome measures	Results		Comments
Bliss et al., 2012 USA [21]	Prospective Cohort	Non-randomized, Non probability consecutive sampling technique.. The study site was conducted at University of Connecticut, Farmington, CT, USA. Data from the American College of Surgeons National Surgical Quality Improvement Program were compared for 2,079 historical control cases, 246 cases without checklist use, and 73 cases with checklist use.		Cases were exposed to checklist usage (n-73), while 2,079 historical controls, 246 without checklist use	Three 60-minute team training sessions were conducted and participants were oriented to the use of a comprehensive surgical checklist. The surgical team used the checklist for high-risk procedures selected from those analyzed for the American College of Surgeons National Surgical Quality Improvement Program. Trained observers assessed the checklist completion and collected data about perioperative communication and safety-compromising events.	Overall completion of the checklist sections was 97.26%. Comparison of 30-day morbidity demonstrated a statistically significant (p = 0.000) reduction in overall adverse event rates from 23.60% for historical control cases and 15.90% in cases with only team training, to 8.20% in cases with checklist use.		Informed consent was used. No comment was made if there was a lost of follow up.
Jammer et al., 2015 European Countries [22]	Prospective Cohort	Data describing surgical checklist use from a 7 day cohort study of surgical outcomes in 28 European nations. Total number of patients included were 45,591. This was a secondary analysis of the European Surgical Outcome Study (EuSOS) data set. Consecutive sampling		65.7% of the settings selected used the surgical safety checklist while the rest of the settings did not. Both the cohorts were compared on the basis of mortality and complications	This was a secondary analysis of the European Surgical Outcome Study (EuSOS) data set. There were no specific instruction as to how to use the surgical checklist. WHO surgical safety checklist was independently used by the settings involved in the study.	Surgical checklist exposure was associated with lower crude hospital mortality (OR 0.84, CI 0.75–0.94; P=0.002). This effect remained after adjustment for baseline risk factors in a multivariate model (adjusted OR 0.81, CI 0.70–0.94; P<0.005) and strengthened after adjusting for variations within countries and hospitals in a three-level generalized mixed model (adjusted OR 0.71, CI 0.58–0.85; P<0.001).		The study included all kinds of surgery patients. The study had a very adequate sample size and 28 European settings were involved.
Lacassie et al., 2016 Chile [23]	Retrospective Cohort	All surgical encounters (n = 70,639) over the period from January 2005 to December 2012. Propensity scoring (PS) methods (matching and inverse weighting) were used to compare the pre and postintervention period, after controlling for selection bias. Consecutive sampling		A total of 29,250 matched pairs were analysed. Both the groups were compared on the basis of mortality and median length of stay in the hospital	The subjects were compared on the basis of two variables which were hospital mortality rate and the length of stay in the hospital.	Hospital mortality rate was 0.82% before and 0.65% after checklist implementation. The median length of stay was 3 days and 2 days for the pre and post-checklist period, respectively.		This is the first Latin American study reporting a decrease in mortality after the implementation of the WHO Surgical Checklist in adult surgical patients. The sample size is very adequate and the analysis was done by matching which makes this study a very strong candidate for considerations. All kinds of surgical patients were included in this study.
Lepänluoma et al., 2015 USA [24]	Retrospective Cohort	All retrospective cases in the neurosurgery department were included in this study. Consecutive sampling technique was used and all those patients who required reoperations were included as a part of this study. The number of reoperations and the reason for conducted them were compared before and after the implementation of the checklist. Consecutive sampling		A total of 5,418 patients were included in this study. Out of these 2,753 patients were exposed to the checklist and were compared to 2,665 patients who were not exposed to the checklist	The subjects were compared on the incidence of the reoperations took place the various reasons the procedures were conducted for. The overall rate of preventable complication-related neurosurgical reoperations were compared and wound infections rates were compared between the two groups.	The overall rate of preventable complication-related neurosurgical reoperations decreased from 3.3% to 2.0% after the checklist implementation. Wound infections were 46% before and 39% after the checklist. All infection-related reoperations proportioned to all neurosurgical operations (2.5% before vs 1.6% after checklist implementation).		The implementation of the WHO surgical checklist in neurosurgery was associated with a decrease in complication-related reoperations, especially those due to preventable infection complications, the majority of which were wound infections.
Ramsay et al., 2019 Scotland [25]	Prospective Cohort	All admissions to any acute hospital in Scotland between 2000 and 2014 were included. Pre-implementation (3 629 602) and Post-implementation (1 825 709) patients were included. Consecutive sampling		Scottish Patient Safety Programme implementation of surgical checklist in all acute surgical care hospital in Scotland	Peri-operative mortality of the patients and the return to the theatre rates were assessed during the study.	The checklist was associated with a 36.6% relative reduction in mortality (P < 0·001). Mortality rates before implementation were decreasing by 0.003%/year; annual decreases of 0.069 per cent were seen during, and 0.019 per cent after, implementation. No such improvement trends were seen in the non-surgical cohort over this time frame. In the preimplementation interval, the return-to-theatre rate increased 0.002% while during the postimplementation interval, it decreased 0·002%.		Since the implementation of the checklist, as part of an overall national safety strategy, there has been a reduction in perioperative mortality. The sample size of the stud and the methodology was very good too.
De Jager et al., 2018 Australia [26]	Retrospective Cohort	Data from 21,306 surgical procedures, performed over a 5-year time period at a tertiary care centre in Australia where the WHO SSC was introduced in the middle of this period. Consecutive sampling		The specific checklist that was implemented was a checklist adapted from the World Health Organization Surgical Safety Checklist to the Australian setting by the Royal Australasian College of Surgeons in consultation with the Australian and New Zealand acclaimed colleges and societies	Operative mortality, length of hospital stay and rates of readmission within 30 days after discharge were examined.	Postoperative mortality rates decreased from 1.2 to 0.92%, and length of admission decreased from 5.2 to 4.7 days (p = 0.014). The reduction in mortality rates reached significance at the 2–3 years post-implementation period [p = 0.017]. The rate of readmission within 30 days of discharge, major wound disruption and septic shock incidence increased over the time.		Implementation of the WHO SSC was associated with a statistically significant reduction in mortality and length of admission over a 5-year time period. This is the first study demonstrating a reduction in postoperative mortality after the implementation of the checklist in an Australian setting. In this study, a relatively longer period examined, comparative to previous international studies.
Westman et al., 2018 New Zealand [27]	Retrospective cohort	The register was searched for superficial and deep SSIs, deep organ SSIs, infections following orthopaedic implantation, and other surgical infections of 4678 neurosurgical patients operated on between 2007 and 2011. Consecutive sampling		WHO surgical safety checklist	Time from operation to the occurrence of infection and the incidence of surgical site infection (SSI) were noted as a part of this study.	Time from operation to infection was shorter before than after checklist implementation (p = 0.039). The overall incidence of SSIs of all neurosurgical patients did not differ (4.1% and 4.5%, respectively.		Only neurosurgery patients were included in this study. The reduction in early postoperative infection rate along with checklist implementation, but not in the long run indicates the complexity of preventing HAIs in neurosurgical patients and need for a multistep infection control approach.
Qualitative Study								
Authors, date & country and focus of study	Study design	Participants, sampling methodology			Data collection & Analysis	Findings	Comments	
Mayer et al., 2016 UK [28]	Longitudinal Study	Surgical admissions (6714 patients) from March 2010 to June 2011 at 5 academic and community hospitals. Consecutive sampling			Checklist usage was recorded as checklist completed in full/partly. Multilevel modeling was performed to investigate the association between complications/mortality and checklist completion. Consecutive sampling was used.	Checklist completion did not affect mortality reduction, but significantly lowered risk of postoperative complication (16.9% vs. 11.2%), and was largely noticed when all 3 components of the checklist had been completed (odds ratio = 0.57, 95% confidence interval: 0.37–0.87, P < 0.01). Calculated population-attributable fractions showed that 14% (95% confidence interval: 7%-21%) of the complications could be prevented if full completion of the checklist was implemented.	Interpretation of the study was clear with a good adequate sample size. The study included gastrointestinal, urological and orthopedic surgery patients only. Checklist was completed in 67.1% of the patients.	

The studies included in this systematic analysis spanned from 2009 to 2021. A total of three studies were from the United States of America [17,21,24]. Additionally, three studies were included from Australia and New Zealand [20,26,27). From the UK and Scotland, two studies were included [25,28]. Single studies were included from Iran [16], Canada [19], Switzerland [18], and Chile [23]. The study by Jammer et al. included multiple settings from various European countries [22].

Study Designs, Sampling Techniques, and Sample Sizes

All studies followed a convenience sampling technique. Among the 13 selected studies, five were interventional studies (before and after) [16-20]. There was only one longitudinal study [28], while four studies were designed as retrospective cohort studies [23,24,26,27]. Additionally, three studies were prospective cohort studies [21,22,25]. The study with the maximum sample size was conducted by Urbach et al. in Canada, involving 109,341 patients before checklist implementation and 106,360 patients after implementation [19]. The smallest sample size was recorded in the study published by Askarian et al. in Iran, which involved 294 surgical patients [16]. Generally, the studies included in the analysis had large sample sizes.

Participants

All 13 studies included patients scheduled for various types of surgeries. Most studies encompassed all types of surgeries conducted within a specific period [18-23,25,26]. Mayer et al. focused solely on gastrointestinal, urological, and orthopedic surgery patients [28]. Two studies included only neurosurgery patients [24,27]. The Iranian study included patients undergoing gastrointestinal surgery exclusively [16], while Haynes et al. included only patients undergoing non-cardiac surgeries [17].

Study Settings

The studies were conducted in operation theatres and surgical departments of the involved hospitals. Some studies had multi-center interventions involving various hospitals, while others included data from single hospitals. Haynes [17], Urbach [19], Moore [20], Jammer [22], Lacassie [23], Ramsay [25], De Jager [26], and Mayer [28] included data from multiple hospitals, whereas the remaining four studies were single-center studies.

Outcomes

Post-operative mortality: According to Haynes et al., the application of the surgical checklist led to a decrease in mortality from 1.5% to 0.8%, which was statistically significant (p=0.003) [17]. However, Lübbeke et al. reported an increase in mortality rate from 4.3% to 5.8%, which was not statistically significant [18]. Urbach et al. reported a decrease in adjusted risk of death from 0.71% to 0.65% [19]. Moore et al. found significant reductions in ninety-day mortality from 4% to 3% [20]. Jammer et al. reported lower crude hospital mortality associated with surgical checklist exposure (OR: 0.84; p=0.002) [22]. Lacassie et al. reported mortality rates of 0.82% before and 0.65% after checklist implementation, indicating a reduction [23]. Ramsay et al. reported a relative reduction of 36.6% in mortality [25]. Similarly, Jager et al. and Westman et al. reported reductions in mortality rates, while Mayer et al. reported different results [26-28].

Complications: Generally, the studies reported a reduction in complication rates among patients. Askarian et al. reported a decrease from 22.9% to 10% [16]. Haynes et al. also reported a decrease from 11% to 7% [17]. Lübbeke et al. noted a decrease in SSIs from 3.0% to 1.7% [18]. Urbach et al. reported a slight decrease from 3.86% to 3.82%, which was not statistically significant [19]. Moore et al. observed a significant decrease in hospital stay (p<0.001) [20]. Bliss et al. reported a decrease in overall adverse event rates from 23.60% to 15.90% [21]. In neurosurgical cases, Lepänluoma et al. and Westman et al. reported no significant decrease in wound infections and SSIs [24,27]. However, Mayer et al. reported a significant decrease in infection rates (16.9% vs. 11.2%) [28]. Jager et al. and Ramsay et al. reported decreases in return to theatre and readmission rates [25,26].

Bias Analysis 

Low risk of bias (Table 3): A few studies demonstrated low risk of bias due to rigorous methodological approaches. Haynes et al. [17] and Urbach et al. [19] are notable examples. Both studies used well-executed randomization and allocation concealment methods. While blinding of participants and personnel was not feasible due to the nature of the interventions, outcome assessors were blinded, and both reported complete data with all pre-specified outcomes. Bliss et al. [21] also displayed a low risk of bias through a well-managed pre-post design, although blinding was not applicable. Ramsay et al. [25] used an appropriate design and reported complete data despite the infeasibility of blinding.

**Table 3:** Cochrane risk of bias analysis.

Study	Random Sequence Generation	Allocation Concealment	Blinding of Participants and Personnel	Blinding of Outcome Assessment	Incomplete Outcome Data	Selective Reporting
Askarian et al., 2011 [16]	Unclear	Unclear	High	Unclear	Low	Unclear
Haynes et al., 2009 [17]	Low	Low	High	Low	Low	Low
Lübbeke et al., 2013 [18]	Unclear	Unclear	High	Unclear	Low	Unclear
Urbach et al., 2014 [19]	Low	Low	Unclear	Unclear	Low	Low
Moore et al., 2022 [20]	Low	Low	Unclear	Low	Unclear	Unclear
Bliss et al., 2012 [21]	Low	Unclear	Unclear	Unclear	Low	Unclear
Jammer et al., 2015 [22]	Low	Low	Unclear	Unclear	Low	Low
Lacassie et al., 2016 [23]	Low	Low	Unclear	Low	Low	Unclear
Lepänluoma et al., 2015 [24]	Unclear	Unclear	Unclear	Unclear	Low	Unclear
Ramsay et al., 2019 [25]	Low	Low	Low	Low	Low	Low
De Jager et al., 2019 [26]	Low	Low	Low	Low	Low	Low
Westman et al., 2018 [27]	Low	Unclear	Low	Unclear	Low	Unclear
Mayer et al., 2016 [28]	Unclear	Unclear	Low	Unclear	Low	Unclear

Unclear risk of bias: Many studies had an unclear risk of bias, primarily due to insufficient details on randomization, allocation concealment, and blinding. Askarian et al. [16] and Lübbeke et al. [18] lacked clear information on these aspects, although they provided complete data. Similarly, Lacassie et al. [23] and Westman et al. [27] had unclear bias risks due to missing details on randomization and blinding procedures, yet reported comprehensive data without other significant biases. De Jager et al. [26] showed unclear risk due to insufficient details on randomization and blinding but provided complete data with no additional biases.

High risk of bias: Some studies faced a high risk of bias due to their design and methodological limitations. Moore et al. [20] had a high risk of bias because of its retrospective design, lack of blinding, and inapplicability of randomization. Jammer et al. [22] had a high risk due to its observational nature without randomization or blinding. Lepänluoma et al. [24] also faced high risk due to unspecified randomization and blinding procedures, though they reported complete data.

Overall, while some studies are robust with a low risk of bias, others show varying degrees of uncertainty or high risk, reflecting diverse methodological strengths and limitations.

Discussion

When discussing adverse outcomes in post-operative patients, it is essential to consider multiple factors to arrive at a comprehensive assessment. Apart from mortality and complications, aspects such as patients' quality of life indicators such as stress and post-operative pain scores should also be examined. While the studies included in this review addressed mortality, SSIs, wound infections, sepsis, return to theatre rates, and hospital stay times, they did not account for patient-reported quality of life measures. Thus, the systematic review is deficient in this aspect. Nonetheless, the reported outcomes were pivotal in evaluating the impact of surgical safety checklists on patient outcomes.

This systematic review comprised 13 studies: five experimental studies, seven cohort and case-control studies, and one longitudinal study. According to the hierarchy of study design preferences, experimental studies provide relatively stronger evidence compared to others. Regarding the effect of the WHO Surgical Safety Checklist on patient mortality, out of 13 studies, only one reported a relative increase in mortality [18]. Additionally, two studies reported insignificant decreases in mortality [19,23], while the remaining 10 studies reported significant reductions. Lübbeke et al. [18] conducted an experimental study with strong evidence, despite a smaller sample size compared to other studies in this review. Conversely, a retrospective cohort study conducted in Scotland, which included 3,629,602 pre-implementation and 1,825,709 post-implementation patients, reported a substantial 36.6% relative reduction in mortality [25]. Urbach et al. [19], another experimental study with a large sample size, reported an insignificant reduction in mortality. However, experimental studies by Haynes [17], Moore [20], and others consistently reported significant decreases in mortality rates. Notably, Moore's study [20] provided a dose-response relationship analysis, enhancing its significance among studies favoring improved mortality outcomes.

Considering the evidence and prioritizing study designs and sample sizes, this systematic review concludes that the application of the WHO Surgical Safety Checklist reduces mortality rates. Discrepancies in reported results can be attributed to various factors. The GlobalSurg Initiative's analysis noted that high-income countries adopting surgical safety checklists did not show significant improvements in mortality compared to low-income countries [29], echoing significant results reported in studies by Askarian [16] and Ramsay [25]. This disparity underscores the checklist's potential impact, especially in settings where its implementation and adherence vary, suggesting a need for greater advocacy and education in its use, particularly in low- and middle-income countries.

Furthermore, an additional analysis considers the Donabedian approach [30], highlighting the psychological impact of checklist implementation on operating theatre staff. The accountability and vigilance fostered among healthcare professionals by checklist use may influence patient outcomes, raising questions about whether improvements are solely due to checklist protocols or also due to the psychological effects on caregivers [31].

Regarding complication rates, among the 13 selected articles, 10 reported that the WHO Surgical Safety Checklist impacted various post-operative complications. Only one study reported a non-significant change in complication rates (from 3.86% to 3.82%) [19]. As mentioned earlier, variability in checklist outcomes may correlate with the Human Development Index of the country where it is implemented. The remaining nine studies consistently reported reductions in complication rates. Bliss et al. [21] also reported a decrease in overall adverse event rates. In contrast, Lepänluoma [24] and Westman [27] found no significant decrease in wound infections and SSIs, whereas Mayer [28] reported a significant decrease in infection rates (16.9% vs. 11.2%). Similarly, studies by Jager et al. [26] and Ramsay et al. [25] reported decreases in return to theatre and readmission rates, reinforcing the checklist's beneficial impact on reducing complications.

When considering study design and sample size, the Canadian study by Urbach et al. [19] reported no significant difference among experimental studies, despite a large sample size compared to studies from Iran [16] and the USA [17]. While the Canadian study's stance on complication rates was neutral, the Scottish retrospective cohort study favored a decrease in complication rates [25]. Based on the evidence synthesized in this systematic review, it concludes that complication rates are significantly reduced with the application of the WHO Surgical Safety Checklist.

This systematic review has examined the impact of the WHO Surgical Safety Checklist on post-operative outcomes across various surgical units globally. Previous systematic reviews on this topic have been conducted [32-36], but these analyses have become outdated, failing to incorporate significant contributions from the past decade. While a more recent review by Delisle et al. explored international surgical outcomes studies, it included multiple confounding factors [6]. In contrast, this systematic review provides a focused perspective on the WHO Surgical Checklist, incorporating recent research from the past decade.

## Conclusions

In conclusion, this review demonstrates that the application of the WHO Surgical Safety Checklist significantly reduces both mortality rates and complications among patients undergoing surgery. While the review did not explore other potential variables that could influence this pathway, such as the acceptability of the checklist among hospital staff, patient quality of life, quality assurance of the implementation process, and adherence to the checklist, it is evident that a positive relationship exists between checklist implementation and improved patient outcomes.

Based on the findings, we recommend that hospitals consider adopting the WHO Surgical Safety Checklist due to its established benefits for patient safety. However, it is crucial to assess the acceptability of the checklist among staff before implementation. Implementing checklists can be challenging in resource-limited settings, such as those in developing countries, potentially leading to staff burnout and adverse outcomes. Therefore, further research is warranted to evaluate the outcomes of implementing the WHO Surgical Safety Checklist in resource-constrained settings. This research would help tailor implementation strategies to ensure effective and sustainable improvements in surgical care across diverse healthcare settings globally.
